# Rehabilitation after surgery for patellar instability

**DOI:** 10.1002/jeo2.12062

**Published:** 2024-06-16

**Authors:** Trine Hysing‐Dahl, Eivind Inderhaug

**Affiliations:** ^1^ Department of Surgery Haraldsplass Deaconess Hospital Bergen Norway; ^2^ Department of Clinical Medicine University of Bergen Bergen Norway; ^3^ Department of Orthopaedic Surgery Haukeland University Hospital Bergen Norway

**Abbreviations**
BPII: Banff Patellofemoral Instability Instrument
LSI: Leg Symmetry Index
MPFL: medial patellofemoral ligament
MPFL‐RSI: medial patellofemoral ligament—return to sport after Injury
PI: patellar instability
ROM: range of motion
RTS: return to sport

## PATELLAR INSTABILITY (PI) SEVERELY AFFECTS PATIENTS' LIFE AND FUNCTION

Recurrent lateral patellar dislocations represent a painful ordeal with a profound impact on patients' lives [26, 57]. The disorder is associated with a substantial reduction in knee function [9, 27, 61], persistent pain [3, 61], kinesiophobia [53], activity limitations [4, 26, 38, 57, 58, 61] and diminished health‐related quality‐of‐life [4, 6, 28, 38, 47, 61]. Moreover, recurrent dislocations frequently result in early osteoarthritic changes in the patellofemoral joint, leaving patients dealing with both knee instability and pain [2, 22]. While PI has traditionally been seen as a traumatic incident, there has been a change in focus on the internal risk factors that predispose patients to recurrent episodes of instability. Unlike patients with other knee injuries, such as anterior cruciate ligament (ACL) tears, predisposing anatomic factors like patella alta, trochlear dysplasia, patella alta and elevated tibial tuberosity to trochlear groove distance [24] are often observed in patients with PI. This finding underlines the developmental perspective of the condition, which makes it more of a chronic disease than merely recurrent episodes of ‘knee incidents’. Consequently, increased apprehension in simple tasks of daily life and increased awareness of the knee are often more pronounced in patients with PI compared to other knee conditions [26].

## SURGERY FOR SOME, REHABILITATION FOR ALL

The complex aetiology of PI makes it a challenging disorder to manage. The standard of care for first‐time dislocators—without osteochondral fractures or loose fragments—is nonoperative management with extensive exercise therapy [16, 36, 59, 60]. For those with recurrent dislocations, surgery is recommended for compliant patients who experience an unacceptable reduction in activities and are willing to undergo postoperative rehabilitation. With a plethora of surgical approaches, there is an ongoing debate on the choice of techniques—but the current mainstay is an individualised (á la carte) approach addressing each patient's deviating anatomy [14]. Reconstruction of the medial patellofemoral ligament (MPFL) is the cornerstone of our approach and is, therefore, performed in all cases. Surgery to correct anatomical predisposing factors includes tibial tubercle osteotomies, trochleoplasty and other osteotomies of the lower extremity. Tibial tubercle osteotomies are performed to reduce the height of the patella on the femur, improving bony support by allowing the patella to enter the trochlear groove earlier in knee flexion. Likewise, medialisation of the tibial tubercle reduces the lateral vector forces on the patella and yields a better position in the trochlear groove [10]. If a patient displays prominent trochlea dysplasia, trochleoplasty is a preferred treatment option for many surgeons [16, 46, 62]. Other osteotomies of the lower extremities are less frequently indicated. The use of femoral derotational osteotomies in cases of femoral anteversion of >30° [16], is a matter of ongoing debate, and its role in an individualised approach is still unclear [10, 16].

Although surgery provides a structurally more stable patella, some patients continue to grapple with postoperative pain [2, 56], impaired knee function [9, 20, 41, 50, 52], psychological distress and persistent avoidance behaviour [26, 29, 39, 57].

The primary aim of treatment, for both surgery and rehabilitation, is to restore patellar stability, enable normal everyday function and allow participation at the individual patient's desired level of activity [3, 10, 33, 62]. Given that the disorder predominantly affects adolescents and young adults, many seek to return to—or even excel in—previous activities after surgery. Hence, comprehensive rehabilitation, including regular functional testing is important to facilitate patients in achieving their goals [5, 33, 44]. The knowledge base on functional assessment and rehabilitation before and after surgery is, however, sparse [13, 30, 33]. In light of the current knowledge of the biomechanics of the patellofemoral joint, and our experience with surgical techniques, we discuss pre‐ and postoperative rehabilitation including the role of functional assessment in patients with PI.

## REHABILITATION IS STAGE‐BASED AND SHOULD ADDRESS FUNCTIONAL DEFICITS

To deliver relevant and targeted rehabilitation, clinicians need a comprehensive understanding of the anatomy and biomechanics of the lower extremities and extensive expertise in the musculoskeletal field [5, 33]. It has been suggested that impaired core muscle function, weak gluteal muscles and reduced balance prior to surgery might lead to prolonged rehabilitation [3]. Hence, prehabilitation is important to facilitate the best possible outcome and to provide a peaceful knee—no joint effusion, full range of motion (ROM) (active/passive) and optimised quadriceps function—before surgery. Typical impairments after recurrent patellar dislocation include quadriceps strength deficits, anterior knee pain, altered movement patterns, reduced balance and proprioceptive control and lack of confidence in the knee [4, 26, 38, 61]. Prehabilitation should, therefore, include education and information, lower extremity muscle strengthening, balance and neuromuscular training to prepare patients for postoperative rehabilitation and improve the course of treatment in accordance with prehabilitation programmes for other knee injuries [12, 21, 65].

Rehabilitation should factor in biological healing with respect to the surgical procedure. Consequently, profound knowledge of the different surgical approaches is paramount. An example is the distalisation of the tibial tubercle for patella alta—where care must be taken to avoid overloading the osteotomy heavy loads through the quadriceps during the first 8 weeks of healing. Restoring ROM, addressing functional deficits in the whole kinetic chain and strengthening exercises for the lower extremities in combination with balance and neuromuscular training are other important aspects of rehabilitation [3, 5, 33, 35, 40, 42, 44, 54, 62, 72]. Moreover, investigations on accelerated protocols (including no/minimal postoperative bracing and weight‐bearing restrictions) have shown promising results compared to more restrictive protocols [37, 45]. Accordingly, over the years, our approach has, therefore, become less restrictive, focusing on early weight‐bearing and initiation of neuromuscular activation as soon as possible after surgery, with knee bracing *only* if low compliance is expected.

Patients with PI constitute a heterogenic group [7, 55, 68], and rehabilitation should, therefore, be individualised to each patient's functional demands and preferences. An overbridging principle is that rehabilitation takes place in phases—with functional gateway criteria deciding progression to the next level (Table 1) [33, 40, 42, 51, 67]. Whether the patient aims to participate in pivoting sports or have normal daily life functioning, some overall goals of rehabilitation are relevant to all: restore knee function, prevent further instability, rebuild confidence in the knee and optimise long‐term quality‐of‐life [33, 35, 40, 44, 62]. A phase‐based approach provides coherence for both patient and clinician, whereas a lack of individualisation and structure can result in a loss of motivation and dissatisfaction with the rehabilitation programme [5].

**Table 1:** Suggested rehabilitation protocol after patellar stabilising surgery.

Main goals with optimal time frame	Precautions	Exercise recommendations
*Early phase MPFL‐R, 0–6 weeks*		
Full passive knee extension Passive knee flexion of >125° No/minimal pain and effusion Adequate quadriceps control Straight‐leg raise with terminal knee extension (no lag sign) Full weight‐bearing with normal gait mechanics Single‐leg balance ≥15 s	Avoid exercises that position the leg in a seated valgus that stress the MPFL graft	Quadriceps sets (isometric and dynamic contractions) (with NMES if necessary) Heel slides Hip and core strengthening (abduction, sit‐ups) Gluteal sets (isometric and dynamic contractions) Mini squats range 0–50° knee flexion Straight leg raises Information on restrictions, expected timeframes pain and scar management
*Early phase bony procedures* a *0–8 weeks*		
Full passive knee extension Passive knee flexion of ≥90° No/minimal pain and effusion Adequate quadriceps control Full weight‐bearing with normal gait mechanics Single‐leg balance ≥15 s	Restricted weight‐bearing 6 weeks Avoid open chain quadriceps exercises to avoid pull at osteotomy site through patellar tendon	Quadriceps sets (isometric and dynamic contractions) (with NMES if necessary) Heel slides Hip and core strengthening (abduction, sit‐ups) Gluteal sets (isometric and dynamic contractions) After 4–6 weeks: ‐ Mini squats range 0–50° knee flexion Information on restrictions, expected timeframes, pain and scar management
*Intermediate phase, 6/8–12 weeks*		
Full ROM No patellar apprehension No effusion or pain after training Adequate muscular control with squats/lunges Satisfactory control with squats/lunges Adequate mechanics in step‐up with full weight‐bearing and terminal knee extension control Single‐leg balance with knee flexed to 30° for >15 s Increase cardiovascular fitness	Avoid open chain quadriceps exercises through large arcs of motion if bony procedures are performed	Quadriceps stretching Progressive resisted quadriceps strengthening (squats, step‐ups, lateral step downs, leg press, wall slides, blood‐flow restriction training) Progressive hamstring, hip and core Strengthening, single‐leg balance exercises Address avoidance behaviour Nonimpact cardiovascular training (bike, elliptical) Information about return to different activities
*Late phase, 12 weeks–6 months*		
Tolerates functional progressions without exacerbation of symptoms Adequate neuromuscular control including adequate landing mechanics with single‐leg hop tests (hop for distance, triple hop, crossover, 6 m timed) Patients with unilateral instability: Quadriceps and hamstring LSI > 80% Hip abductors LSI > 90% Hop tests LSI > 80%	Evaluate potential apprehension in knee‐demanding activities	Maintain ROM and monitor effusion Continue with progressive resisted strengthening Initiate return to jogging programme Advanced plyometrics from double to single‐leg, from simple to complex tasks Advance single‐leg perturbation training Linear change in direction drills Lateral change in direction drills Graded exposure to challenging tasks Sport‐specific cardiovascular training Longer runs (20–30 min in duration)
*RTS phase, 6 months+*		
Complete a test battery assessing physical and mental readiness for RTS If bony procedures are performed; radiographic confirmation of bone healing	A slower recovery and longer RTS time may be expected after bony procedures	Resisted strength training Sport‐specific agility drills Interval training (tempo runs, shuttle runs)

Abbreviations: LSI, limb symmetry index; MPFL‐R, medial patellofemoral ligament reconstruction; NMES, neuromuscular electrical stimulation; ROM, range of motion; RTS, return to sport;

^a^ Brace‐free rehabilitation, except if low compliance is expected.

Throughout the entire rehabilitation, independent of what phase patients are in, education and advice are important to improve self‐efficacy, enhance confidence in the knee and reduce avoidance behaviour. Postoperative restrictions and expected timeframes are part of this education. In addition to pain and scar management.

**Figure 1:**
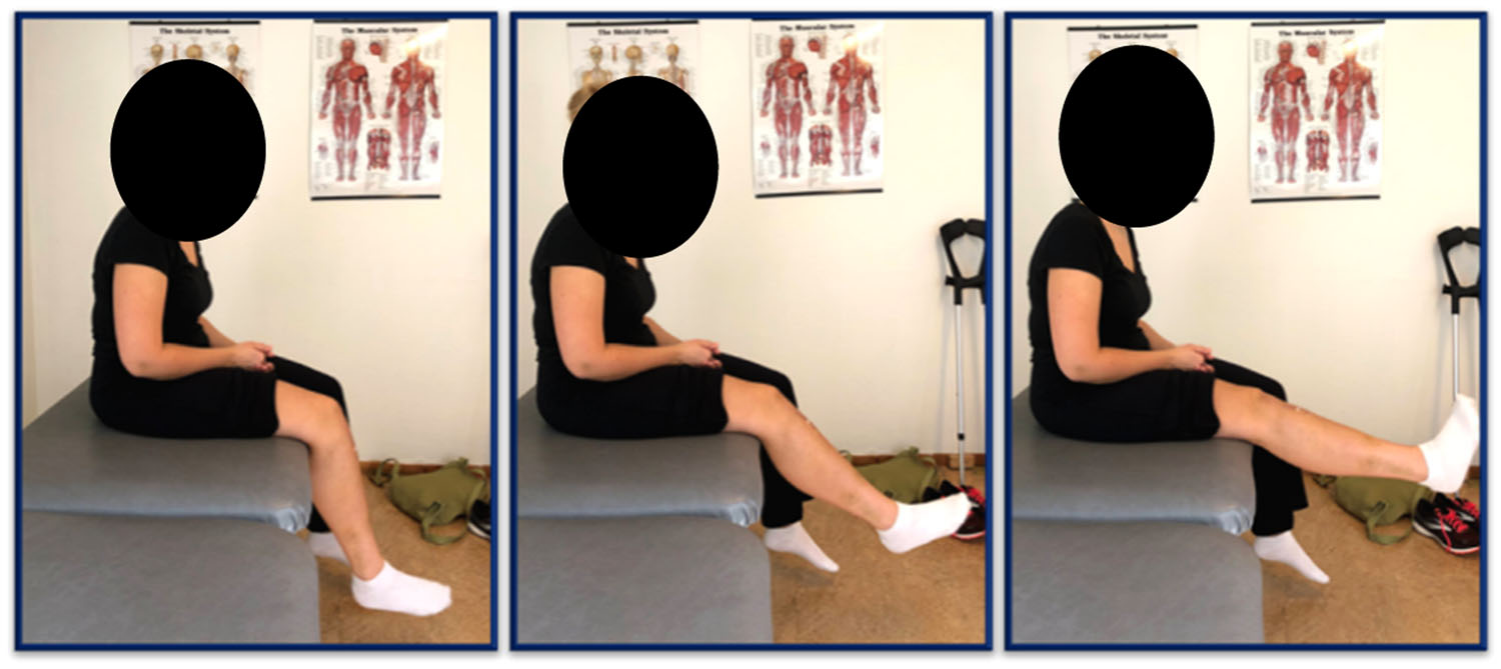
Quadriceps sets, photo: Trine Hysing‐Dahl.

Progression from one phase to the next is based on a compromise between functional recovery, healing from surgery and postoperative pain and effusion. Four to five phases are suggested in the literature [40, 42, 51, 67], where the first *early* phase typically lasts at least 6 weeks (Table 1). The focus in this phase is on protecting the postsurgical knee, reducing pain and swelling, quadriceps activation and restoration of ROM and weight‐bearing within the given restrictions. Quadriceps sets (Figure 1), heel slides and hip and core strengthening constitute relevant exercises. Interventions that position the leg in a seated valgus or similar positions, which stress medial knee tissues should be avoided in this phase to prevent stretching of the reconstructed medial patellofemoral graft [42, 51]. Clinical milestones for safe progression to the next phase include adequate quadriceps control, full passive knee extension and passive knee flexion >125° (90° if bony procedures are performed), effortless full weight‐bearing, no or minimal pain and swelling and normal gait mechanics.

**Figure 2:**
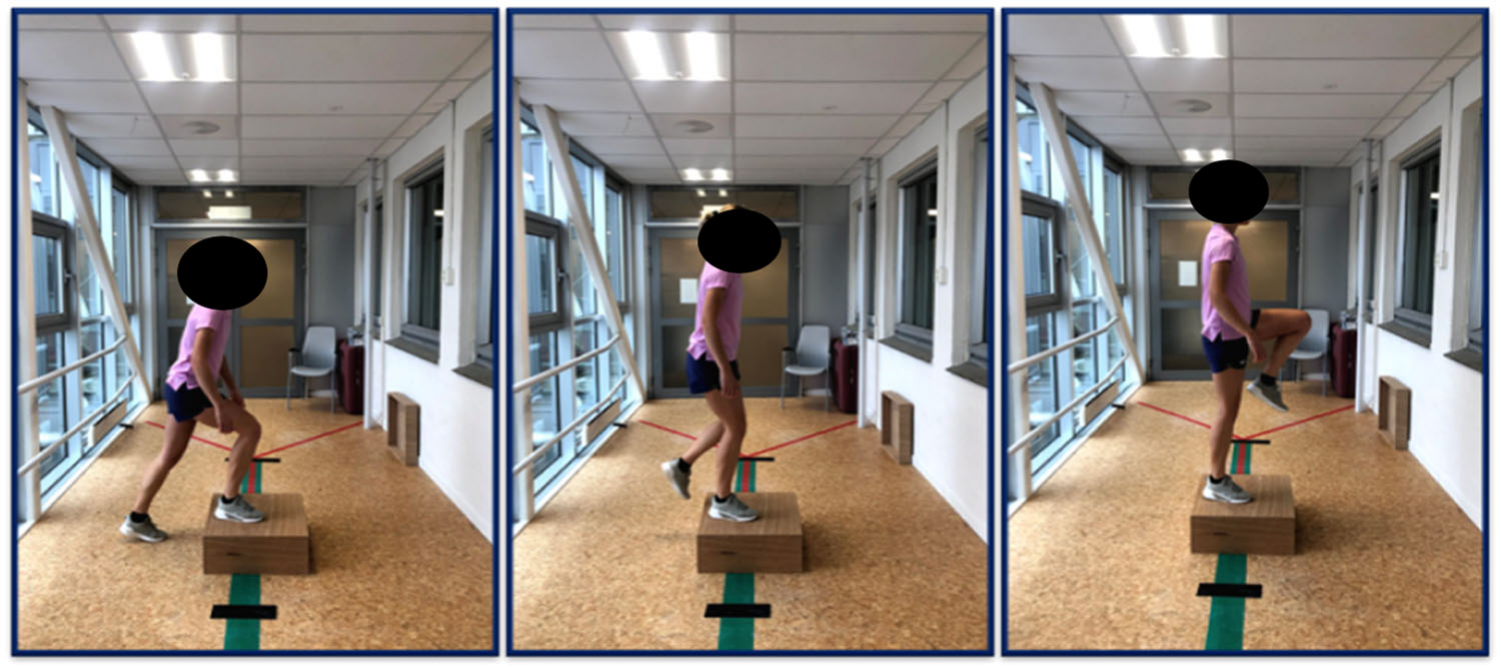
Step‐up task with focus on single‐leg balance and terminal knee extension, photo: Trine Hysing‐Dahl.

In the *intermediate* phase, the focus shifts to progressive strengthening of the lower extremities, functional movement patterns, balance and proprioceptive exercises. The ‘+2 principle’* [18] can be utilised to secure sufficient load progression. Additionally, unilateral strengthening is important to ensure sufficient loading in both the leg that has undergone surgery and the uninvolved leg. Nonimpact cardiovascular training is introduced during this phase. Relevant exercises are step‐up tasks (Figure 2), squats and the use of an elliptical trainer. Blood‐flow restriction training can also be introduced towards the end of this phase to improve quadriceps and hip strength without the need for high‐intensity resistance exercises [11].

Advancement to the *late* phase is due when full ROM is achieved, patients have no pain, effusion or patellar apprehension, and increased hip, hamstrings and quadriceps strength—including improved balance and neuromuscular control.

In the *late* phase, patients continue with strength and proprioceptive training according to principles from the American College of Sports Medicine [[1], p. 464] emphasising sufficient exercise load. Throughout this phase, higher‐impact exercises such as running and jumping/hopping are introduced. Normal daily functioning will be achieved in this phase, and for some patients, this is the overall goal of rehabilitation. Others, who aim to return to the previous or increased level of sports, will progress further during the *late* phase [27, 51] and enter the *return to sport (RTS)* phase when they tolerate functional progressions without exacerbation of symptoms and have completed functional assessment. A time frame of at least 6 months is suggested before enterin this preparatory phase [27, 51] where the goal is to RTS. It comprises higher‐level rehabilitation, including sports‐specific agility drills and more complex plyometric exercises. To ensure a safe return to full competition, the patient's physical and mental readiness for RTS should be evaluated according to objective standards.

## WHEN ARE PATIENTS READY TO RETURN TO DEMANDING ACTIVITIES AND SPORT?

**Figure 3:**
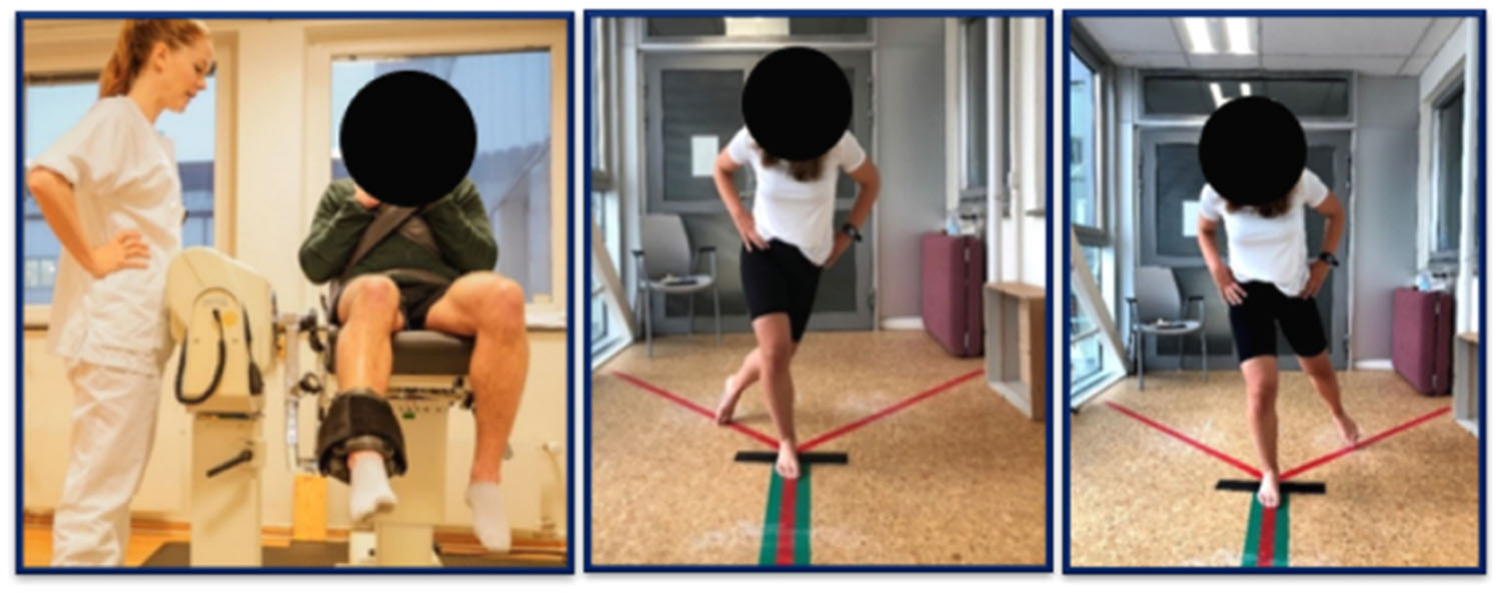
Isokinetic testing and Y‐Balance test, photo: Ingrid Færøyvik and Trine Hysing‐Dahl.

Regular assessment of knee function throughout rehabilitation is important to track progression and inform clinicians when patients are ready to resume knee‐demanding activities. Readiness for return to normal daily activities can be assessed, for example, with a lateral step‐down test [33] and 30 s sit‐to‐stand [63]. Consistent evidence for RTS guidelines in PI patients remains sparse [13, 30, 32]. In our opinion, RTS assessment should comprehensively evaluate all aspects of a patient's knee function—including mental readiness—and comprise both self‐reported and functional measures [32, 43, 70]. Relevant tools that provide information to guide a safe RTS include single‐legged hop tests, side hop tests, Y‐Balance test and isokinetic strength evaluations (Figure 3). However, the test's predictive ability can be affected by oversimplified quantification of performance. For example, hop distance alone does not inform about adequate landing mechanisms and so on. Tests that evaluate the qualitative aspects are, therefore, important [17]. The hop and hold test [64] and single leg squat performed with adequate depth and without significant knee valgus or hip internal rotation [33], are suggested, as both include evaluation of movement quality (e.g., stiff landings and dynamic valgus failure). However, before incorporation, reliable scoring systems need to be developed to accurately capture the *qualitative* aspect of such tests. Other suggestions include two‐dimensional motion analysis of hop tests [15], the Landing Error Scoring System [48] and the qualitative analysis of single‐leg loading [49].

Criteria that suggest physical readiness for RTS include ≤4 cm difference between legs in the Y‐Balance test anterior direction, Leg Symmetry Index (LSI) ≥ 85% on single‐legged hop tests and ≥90% in strength tests [33, 42, 43, 70, 71]. Although regaining dynamic function and strength compared to the uninvolved leg appears to be a reasonable measure, the use of LSI measures seems inappropriate in patients with bilateral instability (up to 50% of all patients undergoing surgery for PI) [27]. Therefore, comparing to age‐ and sex‐matched normative values [66, 69] or evaluating the trajectory of progression over time presents alternative strategies in those patients.

## HOW DO WE MEASURE AND ADDRESS THE PSYCHOLOGICAL ASPECTS OF PI

Unwanted and prolonged psychological responses ought to be evaluated and addressed. How and when clinicians should monitor and address these responses during the treatment course is indeterminate. The MPFL‐RTS after Injury scale [8, 25, 34] and Tampa Scale of Kinesophobia [32, 53] are suggested tools to capture erroneous psychological responses. However, we do not know how accurately they capture the psychological aspects of PI, as none of them have been validated in this patient group.

Suitable alternatives are regular monitoring of mental readiness throughout rehabilitation [19] by use of the validated and diagnose‐specific questionnaire, the Banff Patellofemoral Instability Instrument 2.0 [23]. However, how to interpret BPII 2.0 scores and what values represent mental readiness for RTS are still unclear and need further enlightening before the questionnaire can be of such use.

Interventions to address unwanted psychological responses include information about the disorder, graded exposure to challenging tasks, coping modelling and cognitive‐behavioural therapy where needed. Regular functional testing during rehabilitation can also contribute to increased self‐confidence and restore trust in the knee [31]. Such strategies are more commonly used to address erroneous psychological responses after ACL injuries [19].

## CONCLUSION

The overall goal of rehabilitation after patellar stabilising surgery is to eliminate functional deficits, allowing patients to regain confidence in their knee (physical as well as mental) and enable a return to their desired level of activity. To reach this goal, rehabilitation should be individualised, performed in phases with functional gateway criteria deciding progression to the next phase in addition to factor biological healing and addressing the psychological aspect of PI. Functional assessment with objective criteria should determine patients' mental and physical readiness for returning to sport—although many patients with PI primarily aim to regain normal daily functioning.

## AUTHORS CONTRIBUTION

**T. Hysing‐Dahl**: conception and drafting of manuscript. **E. Inderhaug**: conception and critically revising manuscript.

## CONFLICT OF INTEREST STATEMENT

The authors declare no conflict of interest.

## ETHICS STATEMENT

Ethics approval and consent to participate and Consent for publication is not applicable for this study.

## Footnotes

* When the patient can perform +2 repetitions over the target number of repetitions, load should be increased during the next session.
